# Metabolic modelling of the human gut microbiome in type 2 diabetes patients in response to metformin treatment

**DOI:** 10.1038/s41540-022-00261-6

**Published:** 2023-01-21

**Authors:** Bouchra Ezzamouri, Dorines Rosario, Gholamreza Bidkhori, Sunjae Lee, Mathias Uhlen, Saeed Shoaie

**Affiliations:** 1 Centre for Host–Microbiome Interactions, Faculty of Dentistry, Oral & Craniofacial Sciences, King’s College London, SE1 9RT London, UK; 2 Unit for Population-Based Dermatology, St John’s Institute of Dermatology, King’s College London and Guy’s and St Thomas’ NHS Foundation Trust, London, UK; 3 Science for Life Laboratory, KTH - Royal Institute of Technology, 171 21 Stockholm, Sweden; 4 Present Address: AIVIVO Ltd. Unit 25, Bio-innovation centre, Cambridge Science Park, Cambridge, UK

**Keywords:** Computer modelling, Biochemical networks

## Abstract

The human gut microbiome has been associated with several metabolic disorders including type 2 diabetes mellitus. Understanding metabolic changes in the gut microbiome is important to elucidate the role of gut bacteria in regulating host metabolism. Here, we used available metagenomics data from a metformin study, together with genome-scale metabolic modelling of the key bacteria in individual and community-level to investigate the mechanistic role of the gut microbiome in response to metformin. Individual modelling predicted that species that are increased after metformin treatment have higher growth rates in comparison to species that are decreased after metformin treatment. Gut microbial enrichment analysis showed prior to metformin treatment pathways related to the hypoglycemic effect were enriched. Our observations highlight how the key bacterial species after metformin treatment have commensal and competing behavior, and how their cellular metabolism changes due to different nutritional environment. Integrating different diets showed there were specific microbial alterations between different diets. These results show the importance of the nutritional environment and how dietary guidelines may improve drug efficiency through the gut microbiota.

## Introduction

Type 2 diabetes mellites (T2DM) is a health burden with a rise in epidemic prevalence worldwide^1^. T2D is characterized by increased blood glucose levels (hyperglycemia)^2^. Metformin is the most-prescribed medication to treat patients with T2DM due to its glucose-lowering effects^3^. Metformin propagates insulin sensitivity by mainly reducing hepatic glucose production (through an activation of the hepatic AMP-activated protein kinase protein)^4^. The most common side effect of metformin is gastrointestinal discomfort, including diarrhea, nausea, flatulence and bloating^5^. There is growing evidence in animal and human studies suggesting that the gut microbiome is another target involved in the anti-diabetic effects of metformin^6–9^. Recent investigations documented the therapeutic benefit of orally-administrated metformin compared to intravenously-administrated metformin in T2D patients, suggesting the beneficial contribution of the gut microbiota^10^. Metformin alters the gut microbiome by enhancing *Escherichia sp*, *Akkermansia muciniphila* and *Subdoligranuum variable*; reducing *Intestinibacter bartletti* and increasing the levels of short-chain fatty acids such as butyrate and propionate^7,8^. This could indicate the anti-obesity property of metformin by modulating the gut microbiome and its metabolites. However, the precise mechanisms are unclear.

Understanding the role of bacterial-derived gut metabolites can provide a platform to elucidate interactions between microbe-microbe, microbe-diet and drugs. The gut microbiota is an attractive target for therapeutic intervention and using nutrition may help to promote drug efficiency and reduce gastrointestinal side effects^11^. To elucidate these interactions individual and systems-level analysis is needed^12–14^. Hence, systems biology approaches could be applied to reveal these associations between the abundances of different microbes and the molecular mechanisms underlying metformin treatment on a metabolic level^15^. Genome-scale metabolic models (GEMS) have been used to gain a detailed understanding of microbial metabolic changes in various environments. Previous studies used GEMs to understand the metabolic interactions between microbes and host- microbes^16–18^.

Wu et al. 2017 collected fecal samples from treatment naïve individuals that received 1,700 mg/d metformin treatment (22 patients) for 4 months and generated shotgun metagenomics data to determine the species abundances^7^. In the present study we re-analyzed this metagenomics data with an updated gut microbial gene catalog and metagenome species profile^19^. We carried out further analysis by investigating carbohydrate-active enzymes of the significantly altered species. This analysis showed that species that are decreased after 4 months of metformin treatment have an increased number of annotated mucins and host glycan degradation in comparison to the significantly increased species. In addition, we performed constraint-based analysis using GEMS integrating different diets to predict the phenotype of the drug metformin on the human gut microbiota. These diet-microbiota interactions can help us understand how to increase drug efficiency or mimic drug effects in the gut microbiome of patients with a dysbiosis to an improved phenotype.

## Results

### Gut microbiome profiling

**Fig. 1:**
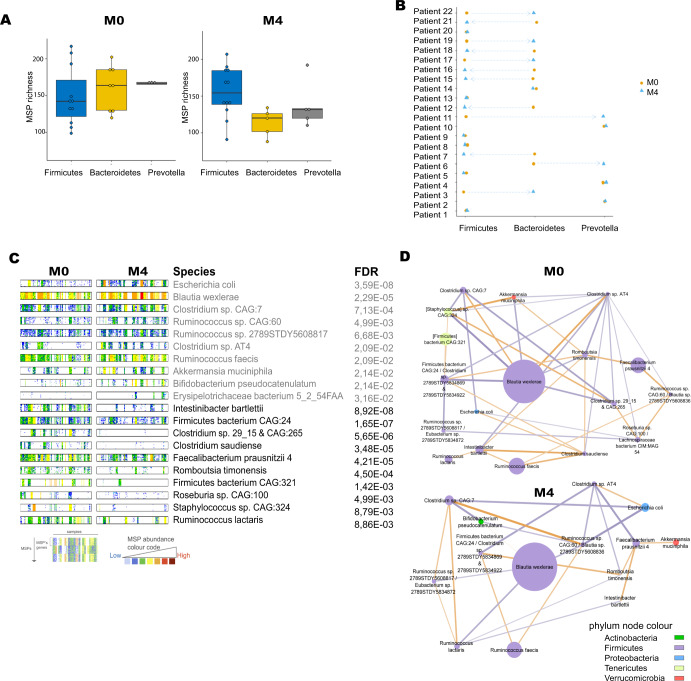
Enterotype and microbial compositional changes. **A** Relation between MSP richness and enterotype before and after metformin treatment (M0 and M4).The boxplots depict minimum and maximum values; centre lines denote mean values; whiskers denote 1.5× the interquartile range; dots denote each patient. **B** Personalized enterotype for each patient before and after metformin treatment (M0 and M4). **C** Barcodes of significantly increased and decreased (Wilcoxon signed-rank test, FDR <0.05) MSPs between M4 and M0. Barcodes represent each sample in the group and respective gene counts of each gene clustered into MSPs. Sample size under analysis: 10 samples that are significantly increased and decreased after metformin treatment (M4). **D** Integrative correlation network (ICN) based on MSPs significance and Spearmen correlation between M4 and M0 (Wilcoxon signed-rank test, FDR <0.05). In the network, the orange edges correspond with a positive correlation whilst the purple edges correspond with a negative correlation. The nodes are coloured based on phylum level whilst the node size correspond with the abundance of each species.

Publicly available metagenomics data of 22 patient with treatment-naïve type 2 diabetes was quantified. In the metagenomics analysis, the latest available human gut microbiome catalog was used to determine the gene abundance. We used metagenome species pangenomes (MSPs) to identify gut microbial species that were perturbed with more in depth functional annotation of key metagenome species (Method). Here, the microbiome composition changed between M0 (before metformin treatment) and M4 (after 4 months of metformin treatment). The significant taxonomic changes on phylum, family and genus level between M0 and M4 can be found in Supplementary table 1. Enterotype analysis was performed using a supervised clustering method with 3 clusters: Bacteroidetes, Firmicutes and Prevotella enterotypes (Fig. 1A). We observed that before metformin treatment (M0) patients were clustered into Bacteroidetes and Firmicutes. After 4 months of metformin treatment (M4) the microbiome changed to Firmicutes and Prevotella enterotype. Personalized analysis of the 22 patients showed how each patient’ enterotype changed after 4 months of metformin treatment (M4) (Fig. 1B). The species-level abundances (Wilcoxon signed-rank test, false discovery rate (FDR < 0.05) of 72 MSPs were significantly different between M4 and M0; 59 MSPs were significantly decreased and 13 significantly increased (Supplementary table 1). Using MSPs and the gut microbial catalog identified additional species with different abundances between M4 and M0 (Fig. 1C). In accordance with the original study, the abundances of *Akkermansia muciniphila, Escherichia coli, Bifidobacterium* were increased whilst the abundance of *Intestinibacter bartlettii* was decreased. We identified additional species - *Blautia wexlerae* (a, short chain fatty acid-producing species) with significantly higher abundances after metformin treatment and several species that had significantly decreased abundances such as *Alistipes obesi (Alistipes genera), Roseburia sp. CAG:100, Faecalibacterium prausnitzii 7, Faecalibacterium prausnitzii 3 (L2–6)*, butyrate producers, and several firmicutes bacteria. To better understand the interactions between the top 10 most significantly increased and decreased classified MSPs we constructed an integrative correlation network (ICN) (Method)^20^. Before metformin treatment many negatively and positively correlated interactions exist between the different species. For instance, *Blautia wexleria* has a positively correlated interaction with the different *Clostridum* species whilst after metformin treatment this is lost. Moreover, after metformin treatment there are less interactions between species. There are 9 species interactions that are common at M0 and M4. At M4 these interactions are stronger between the species. There is one species interaction *between Ruminococcus lactaris* and *Intestinibacter bartlettii* that was positive at M0 and negatively correlated at M4. In addition, most species interactions present at M0 were lost and 16 new species interactions were found at M4 (Fig. 1D; Supplementary table 2).

### Metabolism of carbohydrate was changed with metformin treatment

**Fig. 2:**
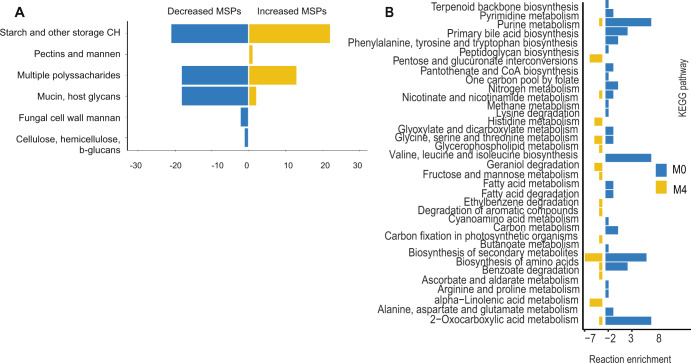
Functional annotation and reaction abundance pathway enrichment. **A** Carbohydrate-active enzymes (CAZymes) genes annotation of the top 10 most significantly increased and decreased species after metformin treatment (Wilcoxon signed-rank test, FDR <0.05). Genes annotated to mucin and host glycans are higher in species that are decreased after metformin treatment. **B** Significant metabolic pathway enrichment based on GEMs for MSPs in 22 patients before (M0) and after treatment (M4) (Wilcoxon signed-rank test, *p* < 0.05).

One of the key factors in the gut microbiome is the metabolism of carbohydrates. CAZymes analysis was performed on genes to identify potential differences in the microbial conversion of carbohydrates (Supplementary table 3). Overall, we found MSPs with increased abundance to be enriched in genes associated with pectins and mannose degradation. Species that were decreased were associated with an increased number of multiple polysaccharides, mucins and host glycan degradation (Fig. 2A). The species *Akkermansia muciniphila* which is well known for degrading host glycans and mucans was significantly increased after metformin treatment.

### GEMS for pathway analysis of associated MSPs

To understand the gut microbiota metabolism after metformin treatment and to reveal interactions between the microbial species and the host, we downloaded the significantly increased and decreased GEM-MSPs with significantly altered abundances between M0 and M4 (https://www.microbiomeatlas.org/). These models were used for personalized community-level metabolic modelling and in depth pathway analysis. For each patient a matrix of reaction abundances before and after metformin treatment was generated based on MSP abundances and reactions that are present in the GEMs. The Kyoto Encyclopedia of Genes and Genomes (KEGG) metabolic pathways was used to identify the differences between the prevalence of metabolic reactions^21^. Personalized metabolic pathway enrichment was performed using the reaction abundance matrix. Through KEGG pathway enrichment analysis, we identified significant alterations before and after metformin treatment (FDR <0.05, Supplementary table 4). Pathways such as phenylalanine, tryptophane and tyrosine biosynthesis were enriched before metformin treatment. Moreover, we found that valine, leucine and isoleucine synthesis was enriched before metformin treatment. After metformin treatment we found enrichment in pathways such as pentose and glucuronate interconversions and histidine metabolism (Fig. 2B).

### GEMS of individual species associated with metformin treatment on various diets

**Fig. 3:**
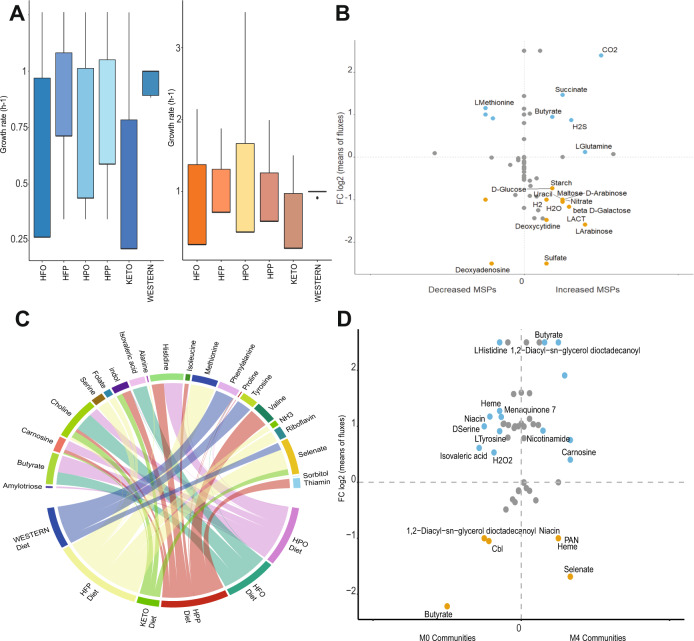
GEM of individual MSPs and personalized gut microbial community modelling. **A** Predictions of individual bacterial growth rate (h-1) constrained on different diets. Overall, MSPs with significantly decreased abundance (boxplots shown in blue) revealed a lower growth rate in comparison to MSPs with significantly increased abundance (boxplots shown in orange). The boxplots depict minimum and maximum values; centre lines denote mean values; whiskers denote 1.5× the interquartile range. **B** Potential contribution to host-intestinal metabolic pool based on metabolites production and consumption of significantly increased and decreased MSPs after metformin treatment (Wilcoxon signed-rank test, FDR < 0.05). The x axis represents the number of decreased or increased MSPs contributing to the metabolite consumption or production in y axis (orange negative or blue positive values, respectively). **C** Personalized gut microbial community modeling of each subject after metformin treatment (M4). Significantly (Wilcoxon signed-rank test, *p* < 0.05) different fluxes (FBA-based predictions) of secreted microbial metabolites on different diets are shown between gut-community models of patients after metformin treatment. The Chord diagram shows the significant means of microbial-metabolites fluxes on each diet. **D** Potential contribution of gut microbiota to host intestinal metabolic pool, based on personalized gut microbial community modeling. Increased secretion of microbial metabolites in control communities (M0) compared to M4 communities are shown in blue; and orange represents the consumed microbial metabolites.

Constrained-based analysis was used for the selected GEM-MSPs. To assess bacterial growth rate and major metabolic activities we used flux balance analysis (FBA) on each MSP-GEM model. Based on the FBA we assessed the potential contributions of bacteria to the gut metabolism. Each model was constrained based on different diets (high fiber omnivorous (HFO), high fiber plant based (HFP), high protein omnivorous (HPO), high protein plant based (HPP), ketogenic (KETO) and western diet (WESTERN.) Overall, the simulations showed lower bacterial growth rate in all diets for species that were decreased in comparison to the increased species after metformin treatment (Fig. 3A). We further analyzed the consumption and production of each metabolite for the increased and decreased species after metformin treatment. There was specific microbial alterations between each diet (Fig. 3B, Supplementary fig. 1, 2). For instance, species that were increased produced a higher amount of butyrate on a HFO diet. Proline was produced by increased species after a HPP diet and KETO diet whilst arginine was produced on a HPO, HPP and HFP diet. Moreover, butyrate and propionate production was linked to decreased species on a HFP and HPO diet whilst increased species produced butyrate on a HFO and HPP diet. Additionally, the simulations showed that NH3 (ammonia) production is more present in species decreased after treatment on a HPP, HPO, Keto, and HFP diet. Increased species produce hydrogen sulphide (H2S) on all diets except the WESTERN diet.

### Metabolic community models show the gut microbial contribution to a changed metabolism after metformin treatment and the importance of diets

The individual microbial activity shows the potential role of each species in the overall gut microbiome metabolism. However, as the microbial species live in communities their behavior changes due to the availability of nutrients and the abundances of the species. Hence, we reconstructed personalized community models for each individual that obtained metformin treatment (22 patients in total) which allowed us to get a deeper insight in the contribution of the gut microbiota to host metabolism. For each personalized community a top 10 of most significantly increased and decreased MSPs per patient was used and different diets were constrained with community biomass as cellular objective. The significant secreted microbial metabolites on different diets before (M0) and after metformin (M4) treatment are shown in Fig. 3C. The personalized community modelling constrained for a HFO diet showed that the metabolites butyrate, carnosine, and nicotinamide (vitamin B3) are produced after metformin treatment (M4) (Fig. 3D). Proline gets produced when constrained on a ketogenic diet as well as valine, and carnosine. There was an increased microbial production of indole and histidine after metformin treatment (M4) on a constrained HPO and HPP diet. On a constrained western diet thiamin is secreted after metformin treatment (M4) (Supplementary fig. 1).

## Discussion

In this study, we performed downstream analysis of the gut microbial abundance alteration before and after metformin treatment using the latest human gut microbiome catalog. This allowed us to identify different gut microbial species and strains. Systems biology approaches have been used to understand the relationship between host and microbiota and to understand biological processes in more details^8,14,16,22^. Here, we used GEMs for the key microbial species that are changed after metformin treatment and performed reaction abundance pathway analysis, individual and community modelling integrating different diets. These analysis gave us insight into the metabolic contribution of the gut microbiota and the effect of metformin treatment in naïve-type2 diabetes patients. We observed increased abundance in *Akkermansia muciniphila, Escherichia coli*, and *Bifidobacterium* whilst the abundance of *Intestinibacter bartlettii* was decreased, which is in agreement with the original study^7,8^. We identified additional species such as *Blautia wexlerae* (a short chain fatty acid producing genus) with significantly higher abundances after metformin treatment and several species that had significantly decreased abundances like *Alistipes obesi* (Alistipes genera), *Roseburia sp. CAG:100, Faecalibacterium prausnitzii 7, Faecalibacterium* and *prausnitzii 3* (L2–6). These decreased species belong to the phyla Firmicutes which is contrary to previous studies showing that Firmicutes are major producers of butyrate and are found to be increased after metformin treatment^23,24^. However, these observed species might be attributed to the differences in strain-specific function. A study in rats demonstrated that metformin treatment changed the intestinal microbiota and showed an increase in the abundance of SCFA-producing Blautia^25^. This corresponds with the increased abundance found for the species *Blautia wexlerae*. Moreover, a cross-sectional study showed that the species *Blautia wexlerae* help reduce inflammation in obesity and insulin resistance^26^. Roseburia has been found to be decreased in metformin-treated patients^27,28^. Also, Faeacalibacterim and Roseburia have been found to be negatively associated with T2D, so it is plausible that metformin treatment decreased these species even more^29^. CAZymes analysis showed that the top 10 most significantly decreased species have mucins and host glycan degradation. Mucins are critical for the maintenance of a homeostatic balanced between the gut microbiota and the host. During mucin degradation, monosacharides and amino acids are released, resulting in nutrients for other gut bacteria. Hence, we expected this phenotype to be present in the increased species after metformin treatment. A possible explanation for this is that the samples present from faeces are different than within the mucosa, which leads to an overrepresentation of bacteria that degrade mucins^30^. A deeper investigation is needed to open new perspectives on the relationship of these species with the host and its effect due to drug treatment. Personalized reaction abundance analysis showed that before metformin treatment the branch chained amino (BCAA) e.g. leucine, valine and isoleucine were increased. Various studies using metabolomics showed that BCAA are elevated in patients with T2D^31,32^. Elevated BCAA can activate the mechanistic target of rapamycin (mTOR) complex 1/ribosomal protein S6 Kinase pathway, which leads to insulin resistance or to a build-up of metabolites that affect the pancreatic islet β-cells^33,34^. After metformin treatment there were no pathways that were enriched in the BCAA suggesting that metformin reduces the circulating levels of BCAA. A study by Sriboonvorakul et al., 2021 showed that BCAA were significantly lower in T2D patients treated with metformin compared to healthy controls^31,35^. A study in mice fed a high fat diet showed that BCAA levels decreased following metformin treatment^36^. Another study, showed how reducing dietary BCAA might be able to restore metabolic health in obese mice consuming a high-fat, high-sugar ‘western diet’^37^. Our modelling showed that isovaleric acid is significantly altered after metformin treatment with a high-fiber omnivorous diet (HFO). Moreover, the individual modelling showed that species decreased after metformin treatment produce BCAA on HFP diet leading to a lower circulation of BCAA. The personalized community modelling showed that before treatment BCAA were produced but not after treatment. Reported side effects of metformin are bloating and intestinal discomfort. These side effects are enhanced by gasses produced by the gut microbiota. Our observations show that species that are increased after metformin treatment produce hydrogen sulphide (H_2_S). Hydrogen sulphide may have several beneficial effects for both host and gut microbiome, such as anti-inflammatory properties and protects the gastrointestinal tract^38^. We also showed that the aromatic amino acid biosynthesis (phenylalanine, tyrosine and tryptophan) was present before metformin treatment. A study by Alqudah et al., 2021 showed that the plasma concentrations of phenylalanine and tryptophane were increased in T2D^39^. This is consistent with the personalized metabolic modelling where we saw a production of tryptophan in a HFP and HPP and western diet. Sugars such as glucose and fructose are one of the most analysed carbohydrates in metabolomics studies and have found to have a positive association with T2D^40,41^. Here, we show that on different diets the species that are increased after treatment consume these sugars, showing that due to metformin the blood sugars levels get decreased so that the human body is more sensitive to insulin. SCFA produced by the gut can lead to beneficial effects on several tissues such as liver, muscle and adipose tissue; thereby improving insulin sensitivity^42^. The ability to produce butyrate is enhanced with patients treated with metformin and can be increased by diet^28,43^. Here, we show with personalized community modelling that butyrate is consumed before metformin treatment and in the individual models that species that are increased after metformin treatment produce butyrate. In a HFO diet butyrate and carnosine is produced after metformin treatment. Carnosine (β-alanyl-L-histidine) has been suggested to improve insulin sensitivity in type 2 diabetes patients, and a growing evidence of animal studies indicate a protective role of carnosine in diabetes^44–46^. Overall, our modelling observations give us insight into how bacteria can have commensal and competing behaviours and how different diets leads to the production and consumption of extracellular metabolites such as SCFA, sugars, aromatic amino acids and BCAA. Understanding how nutritional environments can regulate metformin through the microbiota and elucidating the metabolic pathways may help inform personal dietary guidelines based on the gut microbiota to maximize the drug effect and reduce the gastrointestinal side effects.

## Methods

### Processing and downstream analysis of metformin metagenomics data

Publicly available shotgun metagenomics data on a randomized, placebo-controlled, double-blind type 2 diabetes (T2D) study was downloaded from the sequence read archive under the accession: PRJNA361402^7^. This study consisted of 40 individuals that were treated with either a placebo or metformin. The samples used in this study were samples belonging to the individuals that were treated at baseline (M0) and after 4 months of metformin treatment (M4). The metagenomics cohort was composed by Illumina NextSeq 500 paired end sequencing runs. In this study, Meteor^47^ was used to generate a gene abundance profiling table. Reads were mapped to the updated gut microbial gene catalog and a gene count table for each sample was produced^48^. The R Package MetaOMineR was used for the normalization of the gene counts tables and further downstream analysis^49^. As the metagenomics data was affected by variability in sequencing depth, the data was downsized to 4 million reads to reduce this effect as it might bias the downstream analysis. The latest Metagenomic Species Pan-genomes (MSPs) profile was used to determine the metagenome species for each sample. The abundance of the species were calculated using marker genes in each MSP.

### Richness and enterotype

To study the samples’ bacterial diversity, the MSP richness was obtained based on the MSPs abundance table.

The gene richness is highly sensitive to sequencing depth. Therefore, the mean of the 4 million downsized reads was considered as the richness. DirichletMultinomial^50^ for Clustering and Classification of Microbiome Data was used to identify enterotypes of the samples by defining three components to model and by providing genera count data.

### Carbohydrate-active enZymes annotation

CAZymes analysis was performed on genes to identify potential differences in the microbial conversion of carbohydrates. Gut catalog genes^19^ respective to MSPs were found to be significantly altered considering metformin dysbiosis (M4 against M0) and annotated to dbCAN2^51^. Based on literature review, substrate conversion was identified from annotated Carbohydrate-Active enZymes (CAZymes)^52–56^.

### Genome-scale metabolic model retrieval and modeling

The updated integrated gene catalog^19^ of the human gut microbiome was used to generate the species-specific MSP-GEMs. These models were manually curated to ensure biological functionality. Details regarding the reconstructions of these models are reported by Bidkhori et al.^57^. The MSP-GEMs models were downloaded from the microbiome atlas^58^. Simulation settings were defined using COBRA Toolbox^59^ to properly adjust the models for the desired biological question, such as dietary constrains and anaerobic conditions. The diets and details of the dietary plans that are integrated with the MSP-GEMs can be found in the MIGRENE toolbox^57^. Predictions were made based on flux balance analysis (FBA), where objective function was defined as biomass.

### Individual modelling simulation

To gain insight in the metabolic capabilities of the individual gut microbes we used individual modelling. For individual modelling the COBRA toolbox was used^59^. The top 10 most significantly increased and decreased MSPs-GEMs were selected for individual metabolic modeling. Diet constrains were performed using flux balance analysis (FBA) with biomass as the cellular objective function. The microbial growth rate, consumption and production by the bacteria on 6 different diets were determined. The diets (high protein omnivorous, high protein plant based, high fiber plant-based, high fiber omnivorous, western diet and ketogenic diet in anaerobic conditions) are present in the MIGRENE toolbox (supplementary table 5) and used in this study for individual modelling. Flux variance analysis was used to look into variability in the consumption and production of metabolites where no variability was found (Supplementary table 6).

### Personalized gut-microbial community metabolic models

To gain insight in the metabolic capabilities of the microbe-microbe interactions we used community modelling. Community models for each patient in the metformin cohort were reconstructed using the MIGRENE toolbox^57^. A maximum of 10 MSPs per community was reconstructed due to computational power requirements and to assure model functionality. The S matrices were combined so that each microbe has their own cellular compartment. Each microbial cell has a compartment which represents the intestinal lumen where metabolites from food ingestion is present. Another compartment is present for secreted microbial-metabolites and remaining food-derived metabolites that are not consumed by microbes. These microbial metabolites can be absorbed and reach blood circulation or excreted and present in human faeces. For each community, each individual bacterium biomass function was constrained on the respective abundance in each specific sample. Community biomass is composed of all the different microbes present in the community. FBA was performed with biomass as the objective function. The communities were constrained based on multiple diets (high protein omnivorous, high protein plant-based, high fiber plant-based, high fiber omnivorous, western diet and ketogenic diet) in anaerobic conditions. Models that had similar number of productions across communities in both groups (M0 and M4) were ignored, in order to identify main contributors to increased or decreased secretions after metformin treatment (M4). Only microbial models with production in at least 3 communities in the group were considered.

### KEGG metabolic pathway analysis based on reaction abundance

The MIcrobial and personalized GEM, REactobiome and community NEtwork modeling (MIGRENE) toolbox (https://github.com/sysbiomelab/MIGRENE)^57^ was used for reaction abundance and pathway enrichment analysis. Microbial reaction abundance per sample was determined based on each identified MSP of which a functional metabolic model could be originated. The reaction abundance matrix consists of the abundance of each MSP and all the reactions of each MSP in the sample. This matrix (n × m; n, number of reaction pool and m, number of MSPs) was converted to a binary matrix and then to a binary vector that shows absent/present of each reaction in the samples; present means the reaction was captured in at least one MSP in the sample. These reactions were backtracked to respective KOs, or Enzyme Commission number (EC number) when KO was not available, which in turn were mapped to KEGG metabolic pathways. Reactions with significantly different abundances were identified by Wilcoxon signed-rank test (FDR < 0.05) for dysbiosis. The metabolic pathway enrichment was performed based on microbial reaction presence retrieved from the metabolic models. Hypergeometric test was applied for pathway enrichment. Significant (FDR < 0.05) enriched pathways were identified using Wilcoxon signed-rank test and Bonferroni correction.

### Integrative correlation networks of significant MSPs

Co-occurrence of microbial species was performed based on significant abundance before and after metformin treatment (M0 and M4, respectively) providing a species correlation network for each condition. Compositionality Corrected by REnormalizaion and Permutation (CCREPE) package was applied for the assessment of significance of general similarity measures in the compositional datasets^60^. Similarity measures are calculated based on bootstrapping and permutation approaches for microbial relative abundances. Here, the similarity measure (sim.score) was defined as the nc.score, which implements a robust detection of species-level association patterns adjusted to metagenomics data by calculating co-variation and co-exclusion patterns. Cytoscape^61^ was used to create the networks, where each node represents a MSP; the size of it is proportional to the relative abundance of the respective MSP. The colour of the node represents the MSP phylum level; link between MSPs is represented if the pooled variance z-test shows FDR <0.05 when accounting for compositionality effect; link colour indicates the type of spearman correlation (orange:positive; purple:negative) between linked MSPs.

### Quantification and statistical analysis

Statistical analysis was performed using R software v 4.0.4. Significant taxonomic alterations between M0 (before metformin treatment) and M4 (after metformin treatment) were tested by Wilcoxon signed-rank test and corrected for multiple testing using Benjamini-Hochberg false discovery rate (FDR), q-values (significance FDR <0.05)^62^, which can be found in Fig. 1. In Fig. 2 the significant different abundances and enriched pathways were identified by Wilcoxon signed rank test and Bonferroni correction (FDR <0.05) for metformin dysbiosis.

## Supplementary information

Supplementary figures

Supplementary table 1

Supplementary figure legend

## Data Availability

No new data was generated as part of this study. The metagenomics sequences were downloaded from the original study repository deposited in the sequence read archive under the accession: PRJNA361402. All metabolic models used in this study are available at https://www.microbiomeatlas.org/downloads.php.

## Code availability

The codes used in this study are available at https://github.com/sysbiomelab.
