# Supplementary material for: Metabolic modelling of the human gut microbiome in type 2 diabetes patients in response to metformin treatment
Source: NPJ Syst Biol Appl. 2023 Jan 21;9:2. doi: 10.1038/s41540-022-00261-6 (PMC9867701; doi:10.1038/s41540-022-00261-6)
Supplement: Supplementary file 3 — Supplementary figure legend [file 41540_2022_261_MOESM3_ESM.docx]

**Supplementary Figure Legend**

**Supplementary figure 1. GEM of individual MSPs and personalized gut microbial community**

**modelling on a western, HFP and Keto diet.**

Left of panel A,B and C: Potential contribution to host-intestinal metabolic pool based on metabolites production and consumption of significantly increased and decreased MSPs after metformin treatment (Wilcoxon signed-rank test, FDR < 0.05). The x axis represents the number of decreased or increased MSPs contributing to the metabolite consumption or production in y axis (orange negative or blue positive values, respectively). Right of panel A, B and C: Potential contribution of gut microbiota to host intestinal metabolic pool, based on personalized gut microbial community modeling. Increased secretion of

microbial metabolites in controls communities compared to M4 communities are shown in blue; and orange represents the consumed microbial metabolites. The heatmaps shows the Z scores of the means of microbial metabolites fluxes.

**Supplementary figure 2. GEM of individual MSPs and personalized gut microbial community**

**modelling on a HFO and HPP diet.**

Left of panel A and B: Potential contribution to host-intestinal metabolic pool based on metabolites production and consumption of significantly increased and decreased MSPs after metformin treatment (Wilcoxon signed-rank test, FDR < 0.05). The x axis represents the number of decreased or increased MSPs contributing to the metabolite consumption or production in y axis (orange negative or blue positive values, respectively). Right of panel A and B: Potential contribution of gut microbiota to host intestinal metabolic pool, based on personalized gut microbial community modeling. Increased secretion of microbial metabolites in controls communities compared to M4 communities are shown in blue; and orange represents the consumed microbial metabolites. The heatmaps shows the Z scores of the means of microbial metabolites fluxes.

**Supplementary Table Legend**

Supplementary Table 1. Taxonomic alterations of gut microbiotas between M4 and M0 by applying Wilcoxon signed-rank test and by correcting the p values for multiple testing applying Benjamini-Hochberg FDR. Significance level FDR < 0.05.

Supplementary Table 2. ICN interactions of species

Supplementary Table 3. Substrate conversion of functional annotated CAZymes.

Supplementary Table 4. KEGG Pathway enrichment

Supplementary Table 5. Constrained diet plans

Supplementary Table 6. Flux variance analysis
